# Endoplasmic Reticulum Stress and Mitochondrial Function in Airway Smooth Muscle

**DOI:** 10.3389/fcell.2019.00374

**Published:** 2020-01-15

**Authors:** Philippe Delmotte, Gary C. Sieck

**Affiliations:** Department of Physiology and Biomedical Engineering, Mayo Clinic, Rochester, MN, United States

**Keywords:** mitofusin, IRE1, XBP1, asthma, inflammation

## Abstract

Inflammatory airway diseases such as asthma affect more than 300 million people world-wide. Inflammation triggers pathophysiology via such as tumor necrosis factor α (TNFα) and interleukins (e.g., IL-13). Hypercontraction of airway smooth muscle (ASM) and ASM cell proliferation are major contributors to the exaggerated airway narrowing that occurs during agonist stimulation. An emergent theme in this context is the role of inflammation-induced endoplasmic reticulum (ER) stress and altered mitochondrial function including an increase in the formation of reactive oxygen species (ROS). This may establish a vicious cycle as excess ROS generation leads to further ER stress. Yet, it is unclear whether inflammation-induced ROS is the major mechanism leading to ER stress or the consequence of ER stress. In various diseases, inflammation leads to an increase in mitochondrial fission (fragmentation), associated with reduced levels of mitochondrial fusion proteins, such as mitofusin 2 (Mfn2). Mitochondrial fragmentation may be a homeostatic response since it is generally coupled with mitochondrial biogenesis and mitochondrial volume density thereby reducing demand on individual mitochondrion. ER stress is triggered by the accumulation of unfolded proteins, which induces a homeostatic response to alter protein balance via effects on protein synthesis and degradation. In addition, the ER stress response promotes protein folding via increased expression of molecular chaperone proteins. Reduced Mfn2 and altered mitochondrial dynamics may not only be downstream to ER stress but also upstream such that a reduction in Mfn2 triggers further ER stress. In this review, we summarize the current understanding of the link between inflammation-induced ER stress and mitochondrial function and the role played in the pathophysiology of inflammatory airway diseases.

## Introduction

Inflammation triggers asthma pathophysiology via pro-inflammatory cytokines such as tumor necrosis factor α (TNFα) and interleukin 13 (IL-13). Two hallmarks of asthma are human airway smooth muscle (hASM) hypercontractility and cell proliferation (James, 2005; Joubert and Hamid, 2005; Black et al., 2012; Prakash, 2013, 2016; Wright et al., 2013a, b; Delmotte and Sieck, 2015). It is likely that with asthma, hASM exists in both hyper-contractile and proliferative states, thus contributing to a thicker, more contractile airway. An emergent theme in this context is the role of inflammation-induced endoplasmic reticulum (ER) stress and mitochondria. Previously, we showed that cytokine exposure increases agonist-induced hASM force and ATP consumption due to an increase in contractile protein expression (Dogan et al., 2017). Initially, the increase in hASM ATP demand is met by increased mitochondrial O_2_ consumption and ATP production, but at the expense of reactive oxygen species (ROS) formation and oxidative stress. There is increasing evidence that mitochondria and the ER, although structurally separate organelles, are functionally interdependent units, which must establish links for normal cellular function, including [Ca^2+^]_cyt_ regulation, energy production and cell proliferation (Hajnoczky et al., 2000; Franzini-Armstrong, 2007; Romagnoli et al., 2007; Liesa et al., 2009; Kornmann and Walter, 2010; Antico Arciuch et al., 2012; Glancy and Balaban, 2012; Dorn and Maack, 2013; Kornmann, 2013; Raturi and Simmen, 2013; van Vliet et al., 2014; Filadi et al., 2017). These links are established through specialized ER-mitochondria encounter structures (ERMES) comprising both ER and mitochondrial transmembrane proteins that provide a tethering force between the two organelles to ensure proximity and communication (Franzini-Armstrong, 2007; Kornmann and Walter, 2010; Dorn and Maack, 2013; Kornmann, 2013; Raturi and Simmen, 2013; van Vliet et al., 2014; Filadi et al., 2017). Mitofusin-2 (Mfn2) is an ERMES component that serves to tether mitochondria to the ER. Mfn2 also serves to fuse mitochondria, which together with other fusion proteins [e.g., Mfn1, optic atrophy 1 (Opa1)] elongate mitochondria making them more filamentous, whereas fission proteins such as dynamin related protein 1 (Drp1) and fission 1 protein (Fis1) act to fragment mitochondria. Together these fusion/fission proteins act to dynamically remodel mitochondria under a variety of conditions (Smirnova et al., 2001; James et al., 2003; Lee et al., 2004; Song et al., 2009; Sheridan and Martin, 2010; Palmer et al., 2011; Ranieri et al., 2013). The tethering of mitochondria to the ER allows mitochondrial proximity to ER Ca^2+^ release sites representing a microdomain of higher [Ca^2+^]_cyt_ (“hotspot” > 2 μM) that is essential for mitochondrial Ca^2+^ influx [by activating the mitochondrial Ca^2+^ uniporter (MCU)] (Raffaello et al., 2012). In the absence of mitochondrial Ca^2+^ buffering, the transient [Ca^2+^]_cyt_ response to agonist stimulation is elevated, thereby enhancing hASM force generation. This review will discuss the link between ER stress, Mfn2 expression, mitochondrial tethering to the ER, mitochondrial Ca^2+^ influx, and mitochondrial function in the context of airway inflammation and potential consequences on ASM hyper-contractile and proliferative states.

## Inflammation and Er Stress in Human Asm

**FIGURE 1:**
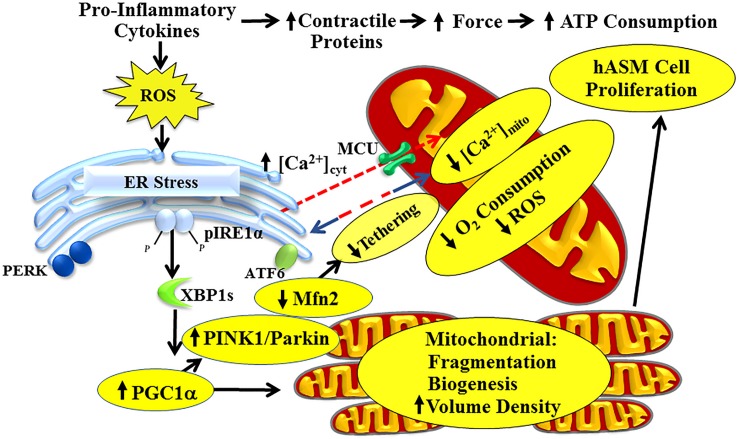
Pro-inflammatory cytokines activate the pIRE1α/XBP1s ER stress pathway in hASM, leading to increased PGC1α and reduced Mfn2 expression. Reduced Mfn2 disrupts tethering of mitochondria to the ER, thereby decreasing mitochondrial Ca^2+^ influx and reducing O_2_ consumption of individual mitochondrion. Increased PGC1α induces mitochondrial biogenesis and increases mitochondrial volume density to meet increased ATP demand. Cytokines also increase hASM force and ATP consumption by increasing contractile protein expression, thereby increasing energetic demand of individual hASM cells. This is mitigated by inducing hASM cell proliferation.

One consequence of inflammation is the unfolding of proteins that accumulate in the lumen of the ER, exposing binding sites for the chaperone protein, binding immunoglobulin protein (BiP). With an accumulation of unfolded proteins, BiP dissociates from three proteins localized at the ER membrane resulting in their activation. The resulting physiological response referred as ER stress or unfolded protein response attempts to restore normal ER function by increasing chaperones proteins expression, halting protein translation and activating protein degradation (Yoshida et al., 2001; Bravo et al., 2012; Garg et al., 2012; Verfaillie et al., 2012; Hasnain et al., 2013; Sano and Reed, 2013; Vannuvel et al., 2013; Delmotte and Sieck, 2015; Kim and Lee, 2015; Zeeshan et al., 2016; Jeong et al., 2017; Navid and Colbert, 2017; Shanahan and Furmanik, 2017; Morris et al., 2018). These three ER stress protein markers involved in this signaling cascade are: protein kinase RNA-like ER kinase (PERK), activating transcription factor 6 (ATF6), and inositol-requiring enzyme 1 (IRE1α, also called serine/threonine-protein kinase/endoribonuclease IRE1α) (Figure 1) (Hai et al., 1989; Nikawa and Yamashita, 1992; Cox et al., 1993; Harding et al., 1999; Li et al., 2000). Phosphorylation of IRE1α (pIRE1α) catalyzes the alternative splicing of XBP1 mRNA (XBP1s) and expression of the active XBP1s transcription factor. Generally, the pIRE1α/XBP1s pathway is associated with upregulation of chaperone protein expression that serves to promote protein refolding and restore ER homeostasis. The RNAse activity of IRE1α is also involved in the regulation of mRNAs through a mechanism called regulated IRE1-dependent decay of mRNA (RIDD) (Hollien and Weissman, 2006). Interestingly, IRE1α could also cleave several pre-miRNAs which could potentially regulate a number of mRNA targets (Upton et al., 2012). As a result, RIDD and therefore, ER stress could affect directly and indirectly a large number of mRNA targets. ATF6 translocates to the Golgi apparatus and is cleaved first by site 1 protease (S1P) and second by site 2 protease (S2P) leading to an active ATF6 transcription factor. As for the pIRE1α/XBP1s pathway, the ATF6 pathway is usually associated with upregulation of chaperone protein expression but also with autophagy, lipid synthesis and endoplasmic-reticulum-associated protein degradation (ERAD) (Yoshida et al., 2001; Bravo et al., 2012; Garg et al., 2012; Verfaillie et al., 2012; Hasnain et al., 2013; Sano and Reed, 2013; Vannuvel et al., 2013; Delmotte and Sieck, 2015; Kim and Lee, 2015; Zeeshan et al., 2016; Jeong et al., 2017; Navid and Colbert, 2017; Shanahan and Furmanik, 2017; Morris et al., 2018). The role of ATF6 in the upregulation of XBP1 and the transcription factor C/EBP homologous protein (CHOP, ER stress-induced apoptosis) is also well documented and reviewed in Hu et al. (2018). Finally, PERK phosphorylates the translation-initiator factor eukaryotic initiation factor 2 (eIF2α), resulting in the translation of activating transcription factor 4 (ATF4). ATF4 is involved in the upregulation of CHOP, ERAD and mitophagy pathways (Adolph et al., 2012; Hasnain et al., 2012, 2013; Dromparis et al., 2013; Kim and Lee, 2015; Zeeshan et al., 2016; Jeong et al., 2017; Navid and Colbert, 2017; Shanahan and Furmanik, 2017; Hu et al., 2018; Morris et al., 2018). In cell types other than hASM, inflammation has been shown to induce ER stress (Adolph et al., 2012; Garg et al., 2012; Hasnain et al., 2012, 2013; Baban et al., 2013; Martino et al., 2013). These studies also demonstrated that the ER stress response is highly variable depending on cell type, species and experimental condition, which subsequently leads to various downstream physiological effects. Inflammation-induced ER stress is most likely a consequence of increased ROS generation (Adolph et al., 2012; Garg et al., 2012; Hasnain et al., 2012, 2013; Baban et al., 2013; Martino et al., 2013), although it is unclear whether ROS is the only mechanism involved. In a recent study, we showed that, TNFα selectively activates the pIRE1α/XBP1s in non-asthmatic hASM cells (Yap et al., 2019). Whether cytokines other than TNFα also selectively activate the pIRE1α/XBP1s pathway is not known. Interestingly, TNFα increases superoxide formation in hASM and Tempol, a superoxide scavenger, reduces the effect of TNFα on the activation of pIRE1α/XBP1s pathway (Yap et al., 2019). To date no other study has explored whether inflammation induces ER stress in hASM and whether an ER stress response in hASM cells from asthmatic patients exists and/or is affected by inflammation. A few studies suggest that the ER stress response is exaggerated in airway epithelial cells or immune cells in the context of asthma (Kim and Lee, 2015; Jeong et al., 2017; Pathinayake et al., 2018). In a mouse model of asthma, chemical chaperones have been used to reduce the ER stress response and attenuate airway hyperresponsiveness (Makhija et al., 2014; Miller et al., 2014; Kim and Lee, 2015; Siddesha et al., 2016).

## Mfn2 and ER Stress Response

In cells other than hASM, the relationship between the ER stress response and mitochondria has gain considerable interest. These previous studies have suggested that Mfn2 and altered mitochondrial dynamics are upstream to ER stress such that a reduction in Mfn2 triggers ER stress (Munoz and Zorzano, 2011; Ngoh et al., 2012; Schneeberger et al., 2013; Bhandari et al., 2015). In a recent study, we suggested that a reduction in Mfn2 in hASM cells is downstream to ER stress (Yap et al., 2019), creating the possibility of a vicious cycle with reduced Mfn2 expression and altered mitochondrial function at the center. Currently, the link between ER stress and downstream regulation of Mfn2 expression has been largely unexplored. A limited number of studies have examined specific downstream targets of activation of the pIRE1α/XBP1s pathway (Calfon et al., 2002; Fonseca et al., 2005; Lipson et al., 2006, 2008; Zeng et al., 2009), but none of these studies have examined effects on Mfn2 expression. As mentioned above, TNFα selectively activates the pIRE1α/XBP1s pathway and reduces Mfn2 expression (Yap et al., 2019), but how IRE1α phosphorylation and XBP1 mRNA splicing affects Mfn2 expression has not been examined in any cell type. Potential targets of interest include peroxisome proliferator-activated receptor gamma coactivator 1-alpha (PGC1α), mitophagy-related proteins PTEN-induced kinase 1 (PINK1) and Parkin, and miRNAs (Figure 1). Several studies found that XBP1s increases expression of PGC1α (Arensdorf et al., 2013; Cheang et al., 2017). Interestingly, PGC1α activates the PINK1/Parkin mitophagy pathway, which is involved in ubiquitination of Mfn2 (Chen and Dorn, 2013; Basso et al., 2018; McLelland et al., 2018). Similarly, a growing list of miRNA has been implicated in the downregulation of Mfn2 but it is not clear if they are expressed in hASM and whether XBP1 is involved in their regulation (Kuhn et al., 2010; Dileepan et al., 2016; Purohit et al., 2019). Previous studies have also shown that XBP1 induces several miRNA but their potential effect on Mfn2 has not been explored and again it’s not known if these miRNA are expressed in hASM (Kuhn et al., 2010; Dileepan et al., 2016; Purohit et al., 2019). The PERK and ATF6 pathway have also been suggested to affect Mfn2 expression, either through PGC1α, mitophagy or ERAD pathways (Morris et al., 2018). It is not known if cytokines other than TNFα activate the PERK and ATF6 pathway in hASM and whether they are activated or amplified in asthmatic hASM. Conversely, the effect of Mfn2 knockdown on IRE1α phosphorylation and XBP1 mRNA splicing has only been examined by four studies – two in neurons, and one each in embryonic fibroblasts and Drosophila (Ngoh et al., 2012; Munoz et al., 2013; Schneeberger et al., 2013; Bhandari et al., 2015).

## Mfn2 and Dynamic Remodeling of Mitochondria

**FIGURE 2:**
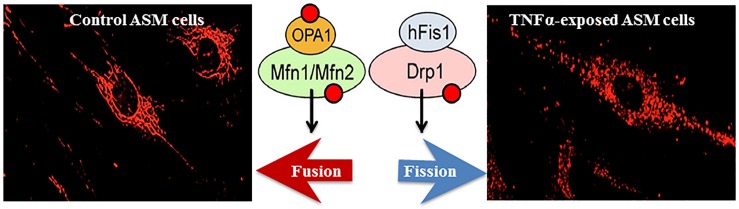
In hASM, TNFα increases mitochondrial fragmentation (fission, visualized using MitoTracker Red); an effect mediated through a reduced expression of fusion proteins (Mfn2, Mfn1, and Opa1) and an increased expression of fission proteins (Drp1 and hFis1).

In hASM, mitochondria are tubular or filamentous but this mitochondria morphology is highly dynamic with mitochondria constantly fusing (fusion) or breaking (fission) from one another. Mitochondria morphology is therefore, the result of this balance between fusion vs. fission (Chen and Chan, 2005; Chan, 2006, 2012; Liesa et al., 2009; Youle and van der Bliek, 2012). This dynamic remodeling is thought to be essential for mitochondrial DNA stability, respiratory function, and to adapt cellular stress resulting from ROS formation (Chan, 2012). Mitochondrial fusion involves the GTPases Mfn1 and/or Mfn2 responsible for the fusion of the outer membrane, and optic atrophy protein 1 (OPA1) for the fusion of the mitochondrial inner membrane (Figure 2). Mfn1 is located only at the mitochondrial outer membrane whereas Mfn2 is localized both at the mitochondrial membrane and in the cytosol. The dimerization of Mfn2 (Mfn2/Mfn2) and/or Mfn1 (Mfn1/Mfn2) tethers outer membranes of neighboring mitochondria (Song et al., 2009; Palmer et al., 2011; Ranieri et al., 2013). Mitochondrial fission depends on the recruitment of cytosolic Drp1 by Fis1 to specific positions around mitochondria and known as constriction sites ultimately leading to fragmentation/fission of the mitochondria (Smirnova et al., 2001; James et al., 2003; Lee et al., 2004; Sheridan and Martin, 2010). The extent of fusion or fission of mitochondria can be quantified using morphological parameters such as form factor (perimeter2/4π×area) and/or aspect ratio (ratio of long and short axis) (Koopman et al., 2005a, b, 2006). We previously reported that Mfn2 expression was reduced in asthmatic hASM, and that this was correlated with an increase in mitochondria fragmentation (Aravamudan et al., 2014). A similar increase in mitochondrial fragmentation in hASM cells was observed after siRNA knockdown of Mfn2 (Aravamudan et al., 2017). In a recent study, we also found that TNFα reduces Mfn2 expression in hASM cells (Yap et al., 2019). As mentioned before, the relation between ER stress and Mfn2 expression and mitochondria fragmentation has been suggested but has not been clearly established.

## Role of Mfn2 in Tethering Mitochondria to ER

There is converging evidence in other cell types that Mfn2 plays an essential role in tethering mitochondria to the ER (Hajnoczky et al., 2002; Patergnani et al., 2011; Raturi and Simmen, 2013; van Vliet et al., 2014; Filadi et al., 2017). Mfn2 located at the ER membrane can dimerize with Mfn2 (Mfn2/Mfn2) and/or Mfn1 (Mfn1/Mfn2) located at the mitochondrial membrane to tether mitochondria to the ER. In hASM cells, a transient [Ca^2+^]_cyt_ response induced by 1 μM ACh stimulation is accompanied by a transient [Ca^2+^]_mito_ response (Delmotte et al., 2012; Delmotte and Sieck, 2015). The transient [Ca^2+^]_mito_ response is blunted by inhibiting the MCU using Ru360. Mitochondrial Ca^2+^ influx via the MCU (Baughman et al., 2011; De Stefani et al., 2011) is only activated by microdomains of higher [Ca^2+^]_cyt_ (“hotspots” > 2 μM) (Gunter et al., 2000; Gunter and Gunter, 2001; Rizzuto and Pozzan, 2006; Gunter and Sheu, 2009; Rizzuto et al., 2009; Giacomello et al., 2010), which exceed the normal global transient [Ca^2+^]_cyt_ response to agonist stimulation in hASM (∼500–600 nM) (Pabelick et al., 1999; Sieck et al., 2008; Sathish et al., 2009, 2011; Delmotte et al., 2012). Higher levels of [Ca^2+^]_cyt_ do occur after 24-h TNFα exposure in response to muscarinic stimulation (Delmotte et al., 2012; Delmotte and Sieck, 2015; Dogan et al., 2017; Sieck et al., 2019), but are still well below levels required to activate the MCU (Gunter et al., 2000; Gunter and Gunter, 2001; Rizzuto and Pozzan, 2006; Gunter and Sheu, 2009; Rizzuto et al., 2009; Giacomello et al., 2010). However, much higher levels of [Ca^2+^]_cyt_ (“hotspots”) are observed in regions in close proximity to the ER Ca^2+^ efflux channels (IP_3_ and RyR). Thus, during agonist stimulation, mitochondria must be tethered to the ER in order to establish close proximity of mitochondria to [Ca^2+^]_cyt_ “hotspots” for mitochondrial Ca^2+^ influx. We previously showed that TNFα disrupts mitochondrial proximity to the ER in hASM cells (Delmotte et al., 2017), but this study only suggests the potential involvement of reduced Mfn2 expression in hASM. Further studies will be necessary to provide direct evidence for the involvement of Mfn2. In hASM cells, it has not been established that Mfn2 is essential for tethering mitochondria to the ER, and thus, for establishing proximity of mitochondria to the ER and microdomains of higher [Ca^2+^]_cyt_ (“hotspots” > 2 μM). Such interactions are cell and context specific, so establishing the role of Mfn2 in hASM is critical. The potential effect of ER stress on microdomains of higher [Ca^2+^]_cyt_, and mitochondrial Ca^2+^ influx is likewise not clearly established.

## Excitation-Energy Coupling Via Mitochondrial Ca^2+^ Influx

**FIGURE 3:**
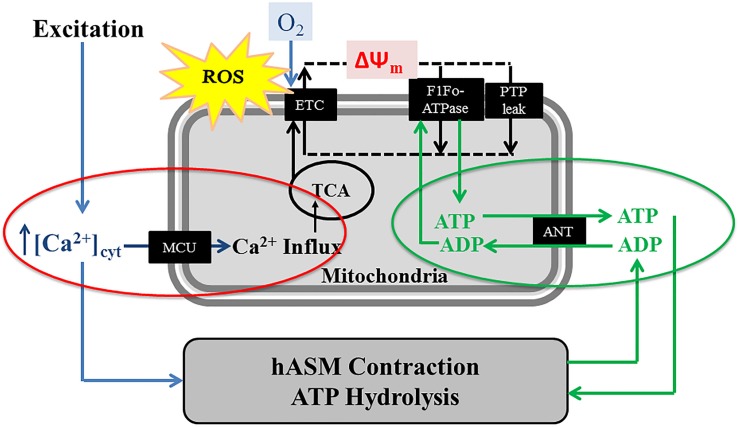
In hASM cells, the agonist-induced transient increase of [Ca^2+^]_cyt_ (excitation) is accompanied by activation of the mitochondrial Ca^2+^ uniporter (MCU) and a transient increase in mitochondrial Ca^2+^ influx, thereby increasing activities of mitochondrial dehydrogenases of the TCA cycle and the electron transport chain (ETC). Mitochondrial O_2_ consumption and ATP synthesis are also linked ATP hydrolysis (and therefore, hASM force) via changes in the ATP/ADP ratio and the adenine nucleotide transporter (ANT). A portion of the O_2_ consumed in the ETC results in ROS formation.

Based on biochemical studies, it is well known that mitochondrial production of ATP (oxidative phosphorylation) depends on dehydrogenase enzyme activities of the tricarboxylic acid (TCA) cycle (or citric acid cycle). Some of these dehydrogenase enzymes [i.e., pyruvate dehydrogenase (PDH), NAD-isocitrate dehydrogenase (ICDH), and oxoglutarate dehydrogenase (OGDH)] are Ca^2+^ dependent (Rizzuto et al., 2000; Parekh, 2003; Franzini-Armstrong, 2007; Maack and O’Rourke, 2007; Romagnoli et al., 2007). Additionally, an increase in [Ca^2+^]_cyt_ stimulates mitochondrial shuttle systems such as the glycerol phosphate shuttle and the aspartate/glutamate transporters resulting in an increase in NADH levels in the mitochondria (Palmieri et al., 2001; Denton, 2009). Thus, mitochondrial Ca^2+^ influx during transient elevation of [Ca^2+^]_cyt_ stimulates dehydrogenase enzyme activities within the TCA cycle and increases, O_2_ consumption, electron transport chain (ETC) flux and ATP production – excitation-energy coupling (Figure 3). Conversely, it is well known that increased ATP consumption leads to transport of ADP into mitochondria via the adenosine nucleotide transporter (ANT), which stimulates ATP synthase (complex V) activity to match ATP production with ATP consumption (Figure 3). Together, these two energy sensing pathways form a normal homeostatic mechanism for excitation-energy coupling in a variety of cell types including hASM. Pathophysiology and mitochondrial dysfunction involve disruptions in these mitochondrial energy-sensing/signaling pathways. As mentioned, most of these studies involved biochemical studies and in some cases isolated mitochondria. While they are critical in our understanding of mitochondrial function, there’s a considerable need for new tools allowing to studies these mechanisms within single mitochondrion in live cells and tissues. A few imaging and/or molecular tools to measure mitochondrial membrane potential ΔΨ_m_, succinate dehydrogenase (SDH) activity (Sieck et al., 1986, 1989, 1995, 1996), ATP consumption (Jones et al., 1999a, b; Dogan et al., 2017), ATP/ADP (Berg et al., 2009), and NAD/NADH ratio (Hung et al., 2011; Cohen et al., 2018) have been developed for use in live cells or tissue but have never been used in hASM.

## Role of ER Stress in Mitochondrial Biogenesis and Increased Mitochondrial Volume Density

A few studies reported that mitochondrial biogenesis in asthmatic hASM is increased but the mechanisms mediating this mitochondrial biogenesis were not explored (Trian et al., 2007; Girodet et al., 2011). These studies also did not examine mitochondria morphology and mitochondrial fragmentation/fission. An increase in mitochondrial volume density is an alternative mechanism to increase ATP production to meet increased ATP demand in hASM after exposure to pro-inflammatory cytokines. In this case, O_2_ consumption in individual mitochondrion can be reduced to minimize formation of ROS. Recent evidence also suggests that reduced Mfn2 and mitochondrial fragmentation is required for mitochondrial biogenesis (Antico Arciuch et al., 2012; Peng et al., 2017), supporting our hypothesis that increased PGC1α and reduced Mfn2 are a coordinated homeostatic response to cytokine-induced activation of the pIRE1α/XBP1s ER stress pathway.

## Cytokine Exposure Increases ROS Generation in hASM

A number of studies have reported that ROS generation increases in asthmatic patients (Katsumata et al., 1990; Comhair and Erzurum, 2010; Zuo et al., 2013) which has the potential to triggers ER stress in hASM. We recently showed exposure of non-asthmatic hASM to TNFα progressively increases superoxide anion formation (Yap et al., 2019). We also found that incubating hASM cells with Tempol (superoxide anion scavenger) mitigated the effects of TNFα in inducing ER stress as well as the reduction in Mfn2 (Yap et al., 2019). It is possible that an increase in ROS generation is triggered, in part by increased ATP consumption and mitochondrial O_2_ consumption. Acute activation of the pIRE1α/XBP1s ER stress pathway leads to a transient reduction in Mfn2 thereby decreasing: (1) mitochondrial tethering to the ER (Figure 1); (2) mitochondrial Ca^2+^ influx (Figures 1, 3); (3) TCA cycle dehydrogenase enzyme activity (Figures 1, 3); (4) O_2_ consumption (Figures 1, 3); and as a result, (5) ROS formation (Figures 1, 3). Without this homeostatic break on mitochondrial O_2_ consumption, the increase in hASM force and ATP consumption induced by pro-inflammatory cytokines will increase ROS formation and further exacerbating ER stress.

## Cytokine Exposure Increases hASM Force, ATP Consumption and Tension Cost

In previous studies, we showed that 24-h exposure of hASM cells to TNFα increases both [Ca^2+^]_cyt_ and force responses to 1 μM muscarinic (ACh) stimulation (White et al., 2006; Sathish et al., 2009, 2011; Delmotte et al., 2012; Jia et al., 2013; Delmotte and Sieck, 2015; Dogan et al., 2017; Sieck et al., 2019). However, hASM sensitivity to muscarinic stimulation is also increased after TNFα, which largely accounts for the enhanced [Ca^2+^]_cyt_ response, but not the force response (Sieck et al., 2019). In recent studies, we found that the increase in ASM force induced by 24-h TNFα exposure is due to an increase in contractile protein expression (Dogan et al., 2017; Sieck et al., 2019). Importantly, the increase in hASM force generation induced by TNFα exposure is associated with an increase in ATP consumption and tension cost (Dogan et al., 2017). This study used an NADH-linked fluorescence technique in permeabilized hASM in which the level of Ca^2+^ activation and force generation can be controlled. In previous studies, we showed that in ASM force generation is directly related to ATP hydrolysis rate (Jones et al., 1999a, b; Dogan et al., 2017). During isometric activation of hASM, ATP hydrolysis rate is initially faster and then declines with time to a sustained level even though isometric force is maintained (the “latch” state). Thus, there is a time-dependent decline in both ATP hydrolysis rate and tension cost that is likely due to cytoskeletal remodeling (Jones et al., 1999b). When actin polymerization in hASM is inhibited, force decreases while ATP hydrolysis rate increases; thereby increasing tension cost (Jones et al., 1999a, b). Normally, tension cost of hASM is dramatically lower than skeletal muscle, but work efficiency is remarkably high (Sieck et al., 1998). Thus, the energetics of hASM are perfectly suited to sustain force at low energy cost. In hASM cells, an increase in ATP consumption is met by stimulation of ATP synthase activity (complex V) and an increase in O_2_ consumption and ATP production (Figures 1, 3). However, stimulation of mitochondrial O_2_ consumption results in increased ROS formation that can trigger protein unfolding and an ER stress response. Thus, we propose that the pIRE1α/XBP1s ER stress pathway represents a homeostatic response directed toward reducing O_2_ consumption and ROS formation in an individual mitochondrion, while increasing mitochondrial biogenesis and mitochondrial volume density to meet the increase in ATP demand. This leads to the question of how energy demand and supply are matched with continued exposure to pro-inflammatory cytokines. Sustained contractility at reduced tension cost is a hallmark of smooth muscle function, and any perturbation should be met with a homeostatic response. One possibility is that hASM cell proliferation (hyperplasia response) provides a mechanism to maintain contractility but at reduced ATP cost per cell.

## Role of Mfn2 in hASM Cell Proliferation

Recent evidence suggests that Mfn2 affects several pro-proliferative pathways and that dynamic mitochondrial remodeling (balance between fusion and fragmentation/fission) governs cell proliferation (Liesa et al., 2009; Antico Arciuch et al., 2012). During cell division, the number of mitochondria or therefore mitochondrial biogenesis needs to increase so each subsequent cell has a similar number of mitochondria (Antico Arciuch et al., 2012). As a result, mitochondria fuse then fragment to generate more mitochondria. Two studies in vascular smooth muscle show that Mfn2 is critical in cell division (Liesa et al., 2009; Antico Arciuch et al., 2012). Notably, the authors show that Mfn2 interacts with several pro-proliferative kinases such as extracellular signal-regulated kinase (ERK1/2) and participates in their inactivation (Liesa et al., 2009; Antico Arciuch et al., 2012). As a result, overexpression of Mfn2 in vascular smooth muscle inhibits cell proliferation (Liesa et al., 2009; Antico Arciuch et al., 2012). Importantly, ERK1/2 activation is believed to play a critical role in hASM proliferation induced by inflammatory cytokines (Lee et al., 2001; Kip et al., 2005; Yu et al., 2013; Dragon et al., 2014; Movassagh et al., 2014). While the relation between ER stress and Mfn2 is not clearly established, studies suggest that ER stress induces cell proliferation in many cell types (Vandewynckel et al., 2013; Chen et al., 2018). Whether ER stress induces hASM cell proliferation is unknown.

## Therapeutic Approaches Targeting Er Stress

An increase in ROS generation is likely responsible for inflammation-induced ER stress. Based on increased ROS generation associated with asthma, the benefits of antioxidant therapeutic have been explored (Heffner and Repine, 1989; Bast et al., 1991; Buhl et al., 1996; Jain and Chandel, 2013). However, one of the challenges with antioxidant treatment is specificity both in terms of ROS targeting and localization (extracellular, cytosol or mitochondrial). It is also now recognized that ROS regulate many cellular signaling cascades and have the potential to be more harmful than beneficial. An alternative therapeutic strategy of reducing ER stress in ASM is the use of chemical chaperone. Well tolerated even at high dose, chemical chaperones are effective in reducing ER stress *in vivo*. Bunezile, the US brand name for sodium phenylbutyrate or 4-phenylbutyrate (4-PBA), is currently used for patients with urea cycle disorders. Chemical chaperones such 4-PBA or tauroursodeoxycholic acid (TUDCA) have gained considerable interest as a potential therapy for a number of other diseases including but not limited to cystic fibrosis [national clinical trial (NCT)00590538 for 4-PBA and NCT00004441 for TUDCA], amyotrophic lateral sclerosis (NCT00107770 for 4-PBA and NCT03800524 for TUDCA) and some types of cancer (NCT00006019 for 4-PBA). A recent phase 1 clinical trial for TUDCA in patients with asthma has been initiated (NCT03878654). Studies in mice showed that 4-PBA attenuated airway inflammation and also reduced airway hyperreactivity in mice model of asthma further indicating a promising therapeutic role for chemical chaperones in the pathogenesis of asthma (Hoffman et al., 2013; Kim et al., 2013; Makhija et al., 2014). The effect of 4-PBA or TUDCA on ASM were not examined and it’s not clear how the chemical chaperone achieved its effect, further illustrating the need to better understand how inflammation induces ER stress in hASM.

## Conclusion and Perspectives

Inflammation, airway hyper-contractility and proliferative remodeling are key aspects of airways diseases such as asthma. The role of inflammation-induced ER stress with downstream impact on Mfn2 and mitochondrial function is of particular interest. The ER stress pathways have been implicated in a growing number of downstream effects ranging from cell death to cell survival. Mfn2 is involved in mitochondrial tethering to the ER, mitochondrial Ca^2+^ influx, O_2_ consumption, and ROS formation. Surprisingly, ER stress and Mfn2 have been largely overlooked in hASM. Mitigation of inflammation-induced ER stress in hASM may represent a novel target for therapeutic intervention.

## Author Contributions

PD and GS wrote the manuscript.

## Conflict of Interest

The authors declare that the research was conducted in the absence of any commercial or financial relationships that could be construed as a potential conflict of interest.

## References

[B1] Adolph T. E. Niederreiter L. Blumberg R. S. Kaser A. (2012). Endoplasmic reticulum stress and inflammation. *Dig. Dis.* 30 341–346. 10.1159/000338121 22796794 PMC3423328

[B2] Antico Arciuch V. G. Elguero M. E. Poderoso J. J. Carreras M. C. (2012). Mitochondrial regulation of cell cycle and proliferation. *Antioxid. Redox Signal.* 16 1150–1180. 10.1089/ars.2011.4085 21967640 PMC3315176

[B3] Aravamudan B. Kiel A. Freeman M. Delmotte P. Thompson M. Vassallo R. (2014). Cigarette smoke-induced mitochondrial fragmentation and dysfunction in human airway smooth muscle. *Am. J. Physiol. Lung. Cell Mol. Physiol.* 306 L840–L854. 10.1152/ajplung.00155.2013 24610934 PMC4116419

[B4] Aravamudan B. Thompson M. Sieck G. C. Vassallo R. Pabelick C. M. Prakash Y. S. (2017). Functional effects of cigarette smoke-induced changes in airway smooth muscle mitochondrial morphology. *J. Cell Physiol.* 232 1053–1068. 10.1002/jcp.25508 27474898 PMC5247322

[B5] Arensdorf A. M. Diedrichs D. Rutkowski D. T. (2013). Regulation of the transcriptome by ER stress: non-canonical mechanisms and physiological consequences. *Front. Genet.* 4:256. 10.3389/fgene.2013.00256 24348511 PMC3844873

[B6] Baban B. Liu J. Y. Mozaffari M. S. (2013). Endoplasmic reticulum stress response and inflammatory cytokines in type 2 diabetic nephropathy: role of indoleamine 2,3-dioxygenase and programmed death-1. *Exp. Mol. Pathol*. 94 343–351. 10.1016/j.yexmp.2012.11.004 23219834

[B7] Basso V. Marchesan E. Peggion C. Chakraborty J. von Stockum S. Giacomello M. (2018). Regulation of ER-mitochondria contacts by Parkin via Mfn2. *Pharmacol. Res* 138 43–56. 10.1016/j.phrs.2018.09.006 30219582

[B8] Bast A. Haenen G. R. Doelman C. J. (1991). Oxidants and antioxidants: state of the art. *Am. J. Med* 91 2S–13S. 10.1016/0002-9343(91)90278-90276 1928207

[B9] Baughman J. M. Perocchi F. Girgis H. S. Plovanich M. Belcher-Timme C. A. Sancak Y. (2011). Integrative genomics identifies MCU as an essential component of the mitochondrial calcium uniporter. *Nature* 476 341–345. 10.1038/nature10234 21685886 PMC3486726

[B10] Berg J. Hung Y. P. Yellen G. (2009). A genetically encoded fluorescent reporter of ATP:ADP ratio. *Nat. Methods* 6 161–166. 10.1038/nmeth.1288 19122669 PMC2633436

[B11] Bhandari P. Song M. Dorn G. W. 2nd (2015). Dissociation of mitochondrial from sarcoplasmic reticular stress in *Drosophila* cardiomyopathy induced by molecularly distinct mitochondrial fusion defects. *J. Mol. Cell Cardiol.* 80 71–80. 10.1016/j.yjmcc.2014.12.018 25555803 PMC4346473

[B12] Black J. L. Panettieri R. A. Jr. Banerjee A. Berger P. (2012). Airway smooth muscle in asthma: just a target for bronchodilation? *Clin. Chest. Med.* 33 543–558. 10.1016/j.ccm.2012.05.002 22929101 PMC3431506

[B13] Bravo R. Gutierrez T. Paredes F. Gatica D. Rodriguez A. E. Pedrozo Z. (2012). Endoplasmic reticulum: ER stress regulates mitochondrial bioenergetics. *Int. J. Biochem. Cell Biol.* 44 16–20. 10.1016/j.biocel.2011.10.012 22064245 PMC4118286

[B14] Buhl R. Meyer A. Vogelmeier C. (1996). Oxidant-protease interaction in the lung. Prospects for antioxidant therapy. *Chest* 110(6 Suppl.), 267S–272S. 10.1378/chest.110.6_supplement.267s 8989163

[B15] Calfon M. Zeng H. Urano F. Till J. H. Hubbard S. R. Harding H. P. (2002). IRE1 couples endoplasmic reticulum load to secretory capacity by processing the XBP-1 mRNA. *Nature* 415 92–96. 10.1038/415092a 11780124

[B16] Chan D. C. (2006). Mitochondrial fusion and fission in mammals. *Annu. Rev. Cell Dev. Biol.* 22 79–99. 10.1146/annurev.cellbio.22.010305.104638 16704336

[B17] Chan D. C. (2012). Fusion and fission: interlinked processes critical for mitochondrial health. *Annu. Rev. Genet.* 46 265–287. 10.1146/annurev-genet-110410-132529 22934639

[B18] Cheang W. S. Wong W. T. Zhao L. Xu J. Wang L. Lau C. W. (2017). PPARdelta Is required for exercise to attenuate endoplasmic reticulum stress and endothelial dysfunction in diabetic mice. *Diabetes* 66 519–528. 10.2337/db15-1657 27856609

[B19] Chen H. Chan D. C. (2005). Emerging functions of mammalian mitochondrial fusion and fission. *Hum. Mol. Genet.* 14 R283–R289. 10.1093/hmg/ddi270 16244327

[B20] Chen L. Liu L. Xie Z. Y. Wang F. Sinkemani A. Zhang C. (2018). Endoplasmic reticulum stress facilitates the survival and proliferation of nucleus pulposus cells in TNF-alpha Stimulus by activating unfolded protein response. *DNA Cell Biol.* 37 347–358. 10.1089/dna.2017.4029 29381432

[B21] Chen Y. Dorn G. W. II (2013). PINK1-phosphorylated mitofusin 2 is a Parkin receptor for culling damaged mitochondria. *Science* 340 471–475. 10.1126/science.1231031 23620051 PMC3774525

[B22] Cohen M. S. Stewart M. L. Goodman R. H. Cambronne X. A. (2018). Methods for using a genetically encoded fluorescent biosensor to monitor nuclear NAD<sup/>. *Methods Mol. Biol.* 1813 391–414. 10.1007/978-1-4939-8588-3_26 30097882 PMC6378224

[B23] Comhair S. A. Erzurum S. C. (2010). Redox control of asthma: molecular mechanisms and therapeutic opportunities. *Antioxid. Redox Signal.* 12 93–124. 10.1089/ARS.2008.2425 19634987 PMC2824520

[B24] Cox J. S. Shamu C. E. Walter P. (1993). Transcriptional induction of genes encoding endoplasmic reticulum resident proteins requires a transmembrane protein kinase. *Cell* 73 1197–1206. 10.1016/0092-8674(93)90648-a 8513503

[B25] De Stefani D. Raffaello A. Teardo E. Szabo I. Rizzuto R. (2011). A forty-kilodalton protein of the inner membrane is the mitochondrial calcium uniporter. *Nature* 476 336–340. 10.1038/nature10230 21685888 PMC4141877

[B26] Delmotte P. Sieck G. C. (2015). Interaction between endoplasmic/sarcoplasmic reticulum stress (ER/SR stress), mitochondrial signaling and Ca(2+) regulation in airway smooth muscle (ASM). *Can. J. Physiol. Pharmacol.* 93 97–110. 10.1139/cjpp-2014-2361 25506723 PMC4386727

[B27] Delmotte P. Yang B. Thompson M. A. Pabelick C. M. Prakash Y. S. Sieck G. C. (2012). Inflammation alters regional mitochondrial Ca(2)+ in human airway smooth muscle cells. *Am. J. Physiol. Cell Physiol.* 303 C244–C256. 10.1152/ajpcell.00414.2011 22673614 PMC3423021

[B28] Delmotte P. Zavaletta V. A. Thompson M. A. Prakash Y. S. Sieck G. C. (2017). TNFalpha decreases mitochondrial movement in human airway smooth muscle. *Am. J. Physiol. Lung. Cell Mol. Physiol.* 313 L166–L176. 10.1152/ajplung.00538.2016 28473328 PMC5538870

[B29] Denton R. M. (2009). Regulation of mitochondrial dehydrogenases by calcium ions. *Biochim. Biophys. Acta* 1787 1309–1316. 10.1016/j.bbabio.2009.01.005 19413950

[B30] Dileepan M. Sarver A. E. Rao S. P. Panettieri RA Jr. Subramanian S. Kannan M. S. (2016). microrna mediated chemokine responses in human airway smooth muscle cells. *PLoS One* 11:e0150842. 10.1371/journal.pone.0150842 26998837 PMC4801396

[B31] Dogan M. Han Y. S. Delmotte P. Sieck G. C. (2017). TNFalpha enhances force generation in airway smooth muscle. *Am. J. Physiol. Lung. Cell Mol. Physiol.* 312 L994–L1002. 10.1152/ajplung.00550.2016 28385814 PMC5495949

[B32] Dorn G. W. II Maack C. (2013). SR and mitochondria: calcium cross-talk between kissing cousins. *J. Mol. Cell Cardiol.* 55 42–49. 10.1016/j.yjmcc.2012.07.015 22902320

[B33] Dragon S. Hirst S. J. Lee T. H. Gounni A. S. (2014). IL-17A mediates a selective gene expression profile in asthmatic human airway smooth muscle cells. *Am. J. Respir. Cell Mol. Biol.* 50 1053–1063. 10.1165/rcmb.2012-0267OC 24393021 PMC4068909

[B34] Dromparis P. Paulin R. Stenson T. H. Haromy A. Sutendra G. Michelakis E. D. (2013). Attenuating endoplasmic reticulum stress as a novel therapeutic strategy in pulmonary hypertension. *Circulation* 127 115–125. 10.1161/CIRCULATIONAHA.112.133413 23149668

[B35] Filadi R. Theurey P. Pizzo P. (2017). The endoplasmic reticulum-mitochondria coupling in health and disease: molecules, functions and significance. *Cell Calcium* 62 1–15. 10.1016/j.ceca.2017.01.003 28108029

[B36] Fonseca S. G. Fukuma M. Lipson K. L. Nguyen L. X. Allen J. R. Oka Y. (2005). WFS1 is a novel component of the unfolded protein response and maintains homeostasis of the endoplasmic reticulum in pancreatic beta-cells. *J. Biol. Chem.* 280 39609–39615. 10.1074/jbc.M507426200 16195229

[B37] Franzini-Armstrong C. (2007). ER-mitochondria communication. How privileged? *Physiology* 22 261–268. 10.1152/physiol.00017.2007 17699879

[B38] Garg A. D. Kaczmarek A. Krysko O. Vandenabeele P. Krysko D. V. Agostinis P. (2012). ER stress-induced inflammation: does it aid or impede disease progression? *Trends Mol. Med.* 18 589–598. 10.1016/j.molmed.2012.06.010 22883813

[B39] Giacomello M. Drago I. Bortolozzi M. Scorzeto M. Gianelle A. Pizzo P. (2010). Ca2+ hot spots on the mitochondrial surface are generated by Ca2+ mobilization from stores, but not by activation of store-operated Ca2+ channels. *Mol. Cell* 38 280–290. 10.1016/j.molcel.2010.04.003 20417605

[B40] Girodet P. O. Ozier A. Bara I. Tunon de Lara J. M. Marthan R. Berger P. (2011). Airway remodeling in asthma: new mechanisms and potential for pharmacological intervention. *Pharmacol. Ther.* 130 325–337. 10.1016/j.pharmthera.2011.02.001 21334378

[B41] Glancy B. Balaban R. S. (2012). Role of mitochondrial Ca2+ in the regulation of cellular energetics. *Biochemistry* 51 2959–2973. 10.1021/bi2018909 22443365 PMC3332087

[B42] Gunter T. E. Buntinas L. Sparagna G. Eliseev R. Gunter K. (2000). Mitochondrial calcium transport: mechanisms and functions. *Cell Calcium* 28 285–296. 10.1054/ceca.2000.0168 11115368

[B43] Gunter T. E. Gunter K. K. (2001). Uptake of calcium by mitochondria: transport and possible function. *IUBMB Life* 52 197–204. 10.1080/15216540152846000 11798033

[B44] Gunter T. E. Sheu S. S. (2009). Characteristics and possible functions of mitochondrial Ca(2+) transport mechanisms. *Biochim. Biophys. Acta* 1787 1291–1308. 10.1016/j.bbabio.2008.12.011 19161975 PMC2730425

[B45] Hai T. W. Liu F. Coukos W. J. Green M. R. (1989). Transcription factor ATF cDNA clones: an extensive family of leucine zipper proteins able to selectively form DNA-binding heterodimers. *Genes Dev.* 3 2083–2090. 10.1101/gad.3.12b.2083 2516827

[B46] Hajnoczky G. Csordas G. Madesh M. Pacher P. (2000). The machinery of local Ca2+ signalling between sarco-endoplasmic reticulum and mitochondria. *J. Physiol.* 529(Pt 1), 69–81. 10.1111/j.1469-7793.2000.00069.x 11080252 PMC2270182

[B47] Hajnoczky G. Csordas G. Yi M. (2002). Old players in a new role: mitochondria-associated membranes, VDAC, and ryanodine receptors as contributors to calcium signal propagation from endoplasmic reticulum to the mitochondria. *Cell Calcium* 32 363–377. 10.1016/s0143416002001872 12543096

[B48] Harding H. P. Zhang Y. Ron D. (1999). Protein translation and folding are coupled by an endoplasmic-reticulum-resident kinase. *Nature* 397 271–274. 10.1038/16729 9930704

[B49] Hasnain S. Z. Lourie R. Das I. Chen A. C. McGuckin M. A. (2012). The interplay between endoplasmic reticulum stress and inflammation. *Immunol. Cell Biol.* 90 260–270. 10.1038/icb.2011.112 22249202 PMC7165805

[B50] Hasnain S. Z. Tauro S. Das I. Tong H. Chen A. C. Jeffery P. L. (2013). IL-10 promotes production of intestinal mucus by suppressing protein misfolding and endoplasmic reticulum stress in goblet cells. *Gastroenterology* 144 357.e9–368 e9. 10.1053/j.gastro.2012.10.043 23123183

[B51] Heffner J. E. Repine J. E. (1989). Pulmonary strategies of antioxidant defense. *Am. Rev. Respir. Dis.* 140 531–554. 10.1164/ajrccm/140.2.531 2669581

[B52] Hoffman S. M. Tully J. E. Nolin J. D. Lahue K. G. Goldman D. H. Daphtary N. (2013). Endoplasmic reticulum stress mediates house dust mite-induced airway epithelial apoptosis and fibrosis. *Respir. Res.* 14:141. 10.1186/1465-9921-14-141 24364984 PMC3877992

[B53] Hollien J. Weissman J. S. (2006). Decay of endoplasmic reticulum-localized mRNAs during the unfolded protein response. *Science* 313 104–107. 10.1126/science.1129631 16825573

[B54] Hu H. Tian M. Ding C. Yu S. (2018). The C/EBP Homologous Protein (CHOP) Transcription Factor Functions in Endoplasmic Reticulum Stress-Induced Apoptosis and Microbial Infection. *Front. Immunol.* 9:3083. 10.3389/fimmu.2018.03083 30662442 PMC6328441

[B55] Hung Y. P. Albeck J. G. Tantama M. Yellen G. (2011). Imaging cytosolic NADH-NAD(+) redox state with a genetically encoded fluorescent biosensor. *Cell Metab.* 14 545–554. 10.1016/j.cmet.2011.08.012 21982714 PMC3190165

[B56] Jain M. Chandel N. S. (2013). Rethinking antioxidants in the intensive care unit. *Am. J. Respir. Crit. Care Med*. 188 1283–1285. 10.1164/rccm.201307-1380CP 24117139 PMC3919077

[B57] James A. (2005). Airway remodeling in asthma. *Curr. Opin. Pulm. Med.* 11 1–6. 10.1097/01.mcp.0000146779.26339.d8 15591881

[B58] James D. I. Parone P. A. Mattenberger Y. Martinou J. C. (2003). HFis1, a novel component of the mammalian mitochondrial fission machinery. *J. Biol. Chem.* 278 36373–36379. 10.1074/jbc.M303758200 12783892

[B59] Jeong J. S. Kim S. R. Cho S. H. Lee Y. C. (2017). Endoplasmic Reticulum Stress and Allergic diseases. *Curr. Allergy Asthma Rep.* 17:82 10.1007/s11882-017-0751-759 PMC5683051 29119328

[B60] Jia L. Delmotte P. Aravamudan B. Pabelick C. M. Prakash Y. S. Sieck G. C. (2013). Effects of the inflammatory cytokines TNF-alpha and IL-13 on stromal interaction molecule-1 aggregation in human airway smooth muscle intracellular Ca(2+) regulation. *Am. J. Respir. Cell Mol. Biol.* 49 601–608. 10.1165/rcmb.2013-0040OC 23713409 PMC3824046

[B61] Jones K. A. Lorenz R. R. Prakash Y. S. Sieck G. C. Warner D. O. (1999a). ATP hydrolysis during contraction of permeabilized airway smooth muscle. *Am. J. Physiol.* 277 L334–L342. 10.1152/ajplung.1999.277.2.L334 10444528

[B62] Jones K. A. Perkins W. J. Lorenz R. R. Prakash Y. S. Sieck G. C. Warner D. O. (1999b). F-actin stabilization increases tension cost during contraction of permeabilized airway smooth muscle in dogs. *J. Physiol.* 519(Pt 2), 527–538. 10.1111/j.1469-7793.1999.0527m.x 10457068 PMC2269509

[B63] Joubert P. Hamid Q. (2005). Role of airway smooth muscle in airway remodeling. *J. Allergy Clin Immunol.* 116 713–716. 10.1016/j.jaci.2005.05.042 16159653

[B64] Katsumata U. Miura M. Ichinose M. Kimura K. Takahashi T. Inoue H. (1990). Oxygen radicals produce airway constriction and hyperresponsiveness in anesthetized cats. *Am. Rev. Respir. Dis.* 141(5 Pt 1), 1158–1161. 10.1164/ajrccm/141.5_Pt_1.1158 2131784

[B65] Kim S. R. Kim D. I. Kang M. R. Lee K. S. Park S. Y. Jeong J. S. (2013). Endoplasmic reticulum stress influences bronchial asthma pathogenesis by modulating nuclear factor kappaB activation. *J. Allergy Clin. Immunol.* 132 1397–1408. 10.1016/j.jaci.2013.08.041 24161747

[B66] Kim S. R. Lee Y. C. (2015). Endoplasmic reticulum stress and the related signaling networks in severe asthma. *Allergy Asthma Immunol. Res.* 7 106–117. 10.4168/aair.2015.7.2.106 25729617 PMC4341331

[B67] Kip S. N. Hunter L. W. Ren Q. Harris P. C. Somlo S. Torres V. E. (2005). [Ca2+]i reduction increases cellular proliferation and apoptosis in vascular smooth muscle cells: relevance to the ADPKD phenotype. *Circ. Res.* 96 873–880. 10.1161/01.RES.0000163278.68142.8a 15790956

[B68] Koopman W. J. Verkaart S. Visch H. J. van der Westhuizen F. H. Murphy M. P. van den Heuvel L. W. (2005a). Inhibition of complex I of the electron transport chain causes O2-. -mediated mitochondrial outgrowth. *Am. J. Physiol. Cell Physiol.* 288 C1440–C1450. 10.1152/ajpcell.00607.2004 15647387

[B69] Koopman W. J. Visch H. J. Verkaart S. van den Heuvel L. W. Smeitink J. A. Willems P. H. (2005b). Mitochondrial network complexity and pathological decrease in complex I activity are tightly correlated in isolated human complex I deficiency. *Am. J. Physiol. Cell Physiol.* 289 C881–C890. 10.1152/ajpcell.00104.2005 15901599

[B70] Koopman W. J. Visch H. J. Smeitink J. A. Willems P. H. (2006). Simultaneous quantitative measurement and automated analysis of mitochondrial morphology, mass, potential, and motility in living human skin fibroblasts. *Cytometry A* 69 1–12. 10.1002/cyto.a.20198 16342116

[B71] Kornmann B. (2013). The molecular hug between the ER and the mitochondria. *Curr. Opin. Cell Biol.* 25 443–448. 10.1016/j.ceb.2013.02.010 23478213

[B72] Kornmann B. Walter P. (2010). ERMES-mediated ER-mitochondria contacts: molecular hubs for the regulation of mitochondrial biology. *J. Cell Sci.* 123(Pt 9), 1389–1393. 10.1242/jcs.058636 20410371 PMC2858017

[B73] Kuhn A. R. Schlauch K. Lao R. Halayko A. J. Gerthoffer W. T. Singer C. A. (2010). MicroRNA expression in human airway smooth muscle cells: role of miR-25 in regulation of airway smooth muscle phenotype. *Am. J. Respir. Cell Mol. Biol.* 42 506–513. 10.1165/rcmb.2009-0123OC 19541842 PMC2848741

[B74] Lee J. H. Johnson P. R. Roth M. Hunt N. H. Black J. L. (2001). ERK activation and mitogenesis in human airway smooth muscle cells. *Am. J. Physiol. Lung. Cell Mol. Physiol.* 280 L1019–L1029. 10.1152/ajplung.2001.280.5.L1019 11290527

[B75] Lee Y. J. Jeong S. Y. Karbowski M. Smith C. L. Youle R. J. (2004). Roles of the mammalian mitochondrial fission and fusion mediators Fis1. Drp1 and Opa1 in apoptosis. *Mol. Biol. Cell* 15 5001–5011. 10.1091/mbc.e04-04-0294 15356267 PMC524759

[B76] Li M. Baumeister P. Roy B. Phan T. Foti D. Luo S. (2000). ATF6 as a transcription activator of the endoplasmic reticulum stress element: thapsigargin stress-induced changes and synergistic interactions with NF-Y and YY1. *Mol. Cell Biol.* 20 5096–5106. 10.1128/mcb.20.14.5096-5106.2000 10866666 PMC85959

[B77] Liesa M. Palacin M. Zorzano A. (2009). Mitochondrial dynamics in mammalian health and disease. *Physiol. Rev.* 89 799–845. 10.1152/physrev.00030.2008 19584314

[B78] Lipson K. L. Fonseca S. G. Ishigaki S. Nguyen L. X. Foss E. Bortell R. (2006). Regulation of insulin biosynthesis in pancreatic beta cells by an endoplasmic reticulum-resident protein kinase IRE1. *Cell Metab.* 4 245–254. 10.1016/j.cmet.2006.07.007 16950141

[B79] Lipson K. L. Ghosh R. Urano F. (2008). The role of IRE1alpha in the degradation of insulin mRNA in pancreatic beta-cells. *PLoS One* 3:e1648. 10.1371/journal.pone.0001648 18286202 PMC2241665

[B80] Maack C. O’Rourke B. (2007). Excitation-contraction coupling and mitochondrial energetics. *Basic Res. Cardiol.* 102 369–392. 10.1007/s00395-007-0666-z 17657400 PMC2785083

[B81] Makhija L. Krishnan V. Rehman R. Chakraborty S. Maity S. Mabalirajan U. (2014). Chemical chaperones mitigate experimental asthma by attenuating endoplasmic reticulum stress. *Am. J. Respir. Cell Mol. Biol.* 50 923–931. 10.1165/rcmb.2013-0320OC 24299608

[B82] Martino M. B. Jones L. Brighton B. Ehre C. Abdulah L. Davis C. W. (2013). The ER stress transducer IRE1beta is required for airway epithelial mucin production. *Mucosal. Immunol.* 6 639–654. 10.1038/mi.2012.105 23168839 PMC4031691

[B83] McLelland G. L. Goiran T. Yi W. Dorval G. Chen C. X. Lauinger N. D. (2018). Mfn2 ubiquitination by PINK1/parkin gates the p97-dependent release of ER from mitochondria to drive mitophagy. *Elife* 7:e32866. 10.7554/eLife.32866 29676259 PMC5927771

[B84] Miller M. Rosenthal P. Beppu A. Mueller J. L. Hoffman H. M. Tam A. B. (2014). ORMDL3 transgenic mice have increased airway remodeling and airway responsiveness characteristic of asthma. *J. Immunol.* 192 3475–3487. 10.4049/jimmunol.1303047 24623133 PMC3981544

[B85] Morris G. Puri B. K. Walder K. Berk M. Stubbs B. Maes M. (2018). The endoplasmic reticulum stress response in neuroprogressive diseases: emerging pathophysiological role and translational implications. *Mol. Neurobiol.* 55 8765–8787. 10.1007/s12035-018-1028-1026 29594942 PMC6208857

[B86] Movassagh H. Shan L. Halayko A. J. Roth M. Tamm M. Chakir J. (2014). Neuronal chemorepellent Semaphorin 3E inhibits human airway smooth muscle cell proliferation and migration. *J. Allergy Clin. Immunol.* 133 560–567. 10.1016/j.jaci.2013.06.011 23932461

[B87] Munoz J. P. Ivanova S. Sanchez-Wandelmer J. Martinez-Cristobal P. Noguera E. Sancho A. (2013). Mfn2 modulates the UPR and mitochondrial function via repression of PERK. *Embo. J.* 32 2348–2361. 10.1038/emboj.2013.168 23921556 PMC3770335

[B88] Munoz J. P. Zorzano A. (2011). Endoplasmic reticulum stress enters a Nogo zone. *Sci. Transl. Med.* 3:88s26. 10.1126/scitranslmed.3002708 21697529

[B89] Navid F. Colbert R. A. (2017). Causes and consequences of endoplasmic reticulum stress in rheumatic disease. *Nat. Rev. Rheumatol.* 13 25–40. 10.1038/nrrheum.2016.192 27904144

[B90] Ngoh G. A. Papanicolaou K. N. Walsh K. (2012). Loss of mitofusin 2 promotes endoplasmic reticulum stress. *J. Biol. Chem.* 287 20321–20332. 10.1074/jbc.M112.359174 22511781 PMC3370214

[B91] Nikawa J. Yamashita S. (1992). IRE1 encodes a putative protein kinase containing a membrane-spanning domain and is required for inositol phototrophy in Saccharomyces cerevisiae. *Mo. l Microbiol.* 6 1441–1446. 10.1111/j.1365-2958.1992.tb00864.x 1625574

[B92] Pabelick C. M. Prakash Y. S. Kannan M. S. Sieck G. C. (1999). Spatial and temporal aspects of calcium sparks in porcine tracheal smooth muscle cells. *Am. J. Physiol.* 277 L1018–L1025. 10.1152/ajplung.1999.277.5.L1018 10564188

[B93] Palmer C. S. Osellame L. D. Stojanovski D. Ryan M. T. (2011). The regulation of mitochondrial morphology: intricate mechanisms and dynamic machinery. *Cell Signal.* 23 1534–1545. 10.1016/j.cellsig.2011.05.021 21683788

[B94] Palmieri L. Pardo B. Lasorsa F. M. del Arco A. Kobayashi K. Iijima M. (2001). Citrin and aralar1 are Ca(2+)-stimulated aspartate/glutamate transporters in mitochondria. *Embo. J.* 20 5060–5069. 10.1093/emboj/20.18.5060 11566871 PMC125626

[B95] Parekh A. B. (2003). Mitochondrial regulation of intracellular Ca2+ signaling: more than just simple Ca2+ buffers. *News Physiol. Sci.* 18 252–256. 10.1152/nips.01458.2003 14614159

[B96] Patergnani S. Suski J. M. Agnoletto C. Bononi A. Bonora M. De Marchi E. (2011). Calcium signaling around Mitochondria Associated Membranes (MAMs). *Cell Commun. Signal.* 9:19. 10.1186/1478-811X-9-19 21939514 PMC3198985

[B97] Pathinayake P. S. Hsu A. C. Waters D. W. Hansbro P. M. Wood L. G. Wark P. A. B. (2018). Understanding the Unfolded Protein Response in the Pathogenesis of Asthma. *Front. Immunol.* 9:175. 10.3389/fimmu.2018.00175 29472925 PMC5810258

[B98] Peng K. Yang L. Wang J. Ye F. Dan G. Zhao Y. (2017). The interaction of mitochondrial biogenesis and fission/fusion mediated by PGC-1alpha regulates rotenone-induced dopaminergic neurotoxicity. *Mol. Neurobiol.* 54 3783–3797. 10.1007/s12035-016-9944-9949 27271125

[B99] Prakash Y. S. (2013). Airway smooth muscle in airway reactivity and remodeling: what have we learned? *Am. J. Physiol. Lung. Cell Mol. Physiol.* 305 L912–L933. 10.1152/ajplung.00259.2013 24142517 PMC3882535

[B100] Prakash Y. S. (2016). Emerging concepts in smooth muscle contributions to airway structure and function: implications for health and disease. *Am. J. Physiol. Lung. Cell Mol. Physiol.* 311 L1113–L1140. 10.1152/ajplung.00370.2016 27742732 PMC5206394

[B101] Purohit P. K. Edwards R. Tokatlidis K. Saini N. (2019). MiR-195 regulates mitochondrial function by targeting mitofusin-2 in breast cancer cells. *RNA Biol.* 16 918–929. 10.1080/15476286.2019.1600999 30932749 PMC6546347

[B102] Raffaello A. De Stefani D. Rizzuto R. (2012). The mitochondrial Ca(2+) uniporter. *Cell Calcium.* 52 16–21. 10.1016/j.ceca.2012.04.006 22672876

[B103] Ranieri M. Brajkovic S. Riboldi G. Ronchi D. Rizzo F. Bresolin N. (2013). Mitochondrial fusion proteins and human diseases. *Neurol. Res. Int.* 2013:293893. 10.1155/2013/293893 23781337 PMC3678461

[B104] Raturi A. Simmen T. (2013). Where the endoplasmic reticulum and the mitochondrion tie the knot: the mitochondria-associated membrane (MAM). *Biochim. Biophys. Acta* 1833 213–224. 10.1016/j.bbamcr.2012.04.013 22575682

[B105] Rizzuto R. Bernardi P. Pozzan T. (2000). Mitochondria as all-round players of the calcium game. *J. Physiol.* 529(Pt 1), 37–47. 10.1111/j.1469-7793.2000.00037.x 11080249 PMC2270183

[B106] Rizzuto R. Marchi S. Bonora M. Aguiari P. Bononi A. De Stefani D. (2009). Ca(2+) transfer from the ER to mitochondria: when, how and why. *Biochim. Biophys. Acta* 1787 1342–1351. 10.1016/j.bbabio.2009.03.015 19341702 PMC2730423

[B107] Rizzuto R. Pozzan T. (2006). Microdomains of intracellular Ca2+: molecular determinants and functional consequences. *Physiol. Rev.* 86 369–408. 10.1152/physrev.00004.2005 16371601

[B108] Romagnoli A. Aguiari P. De Stefani D. Leo S. Marchi S. Rimessi A. (2007). Endoplasmic reticulum/mitochondria calcium cross-talk. *Novartis Found. Symp.* 287 122–131. discussion 131–129. 18074635

[B109] Sano R. Reed J. C. (2013). ER stress-induced cell death mechanisms. *Biochim. Biophys. Acta* 1833 3460–3470. 10.1016/j.bbamcr.2013.06.028 23850759 PMC3834229

[B110] Sathish V. Delmotte P. F. Thompson M. A. Pabelick C. M. Sieck G. C. Prakash Y. S. (2011). Sodium-calcium exchange in intracellular calcium handling of human airway smooth muscle. *PLoS One* 6:e23662. 10.1371/journal.pone.0023662 21858195 PMC3156227

[B111] Sathish V. Thompson M. A. Bailey J. P. Pabelick C. M. Prakash Y. S. Sieck G. C. (2009). Effect of proinflammatory cytokines on regulation of sarcoplasmic reticulum Ca2+ reuptake in human airway smooth muscle. *Am. J. Physiol. Lung. Cell Mol. Physiol.* 297 L26–L34. 10.1152/ajplung.00026.2009 19395670 PMC2711800

[B112] Schneeberger M. Dietrich M. O. Sebastian D. Imbernon M. Castano C. Garcia A. (2013). Mitofusin 2 in POMC neurons connects ER stress with leptin resistance and energy imbalance. *Cell* 155 172–187. 10.1016/j.cell.2013.09.003 24074867 PMC3839088

[B113] Shanahan C. M. Furmanik M. (2017). Endoplasmic reticulum stress in arterial smooth muscle cells: a novel regulator of vascular disease. *Curr. Cardiol. Rev.* 13 94–105. 10.2174/1573403X12666161014094738 27758694 PMC5440785

[B114] Sheridan C. Martin S. J. (2010). Mitochondrial fission/fusion dynamics and apoptosis. *Mitochondrion* 10 640–648. 10.1016/j.mito.2010.08.005 20727425

[B115] Siddesha J. M. Nakada E. M. Mihavics B. R. Hoffman S. M. Rattu G. K. Chamberlain N. (2016). Effect of a chemical chaperone, tauroursodeoxycholic acid, on HDM-induced allergic airway disease. *Am. J. Physiol. Lung. Cell Mol. Physiol.* 310 L1243–L1259. 10.1152/ajplung.00396.2015 27154200 PMC4935467

[B116] Sieck G. C. Dogan M. Young-Soo H. Osorio Valencia S. Delmotte P. (2019). Mechanisms underlying TNFalpha-induced enhancement of force generation in airway smooth muscle. *Physiol. Rep.* 7:e14220. 10.14814/phy2.14220 31512410 PMC6739507

[B117] Sieck G. C. Fournier M. Prakash Y. S. Blanco C. E. (1996). Myosin phenotype and SDH enzyme variability among motor unit fibers. *J. Appl. Physiol.* 80 2179–2189. 10.1152/jappl.1996.80.6.2179 8806928

[B118] Sieck G. C. Han Y. S. Prakash Y. S. Jones K. A. (1998). Cross-bridge cycling kinetics, actomyosin ATPase activity and myosin heavy chain isoforms in skeletal and smooth respiratory muscles. *Comp. Biochem. Physiol. B Biochem. Mol. Biol.* 119 435–450. 10.1016/s0305-0491(98)00005-4 9734328

[B119] Sieck G. C. Lewis M. I. Blanco C. E. (1989). Effects of undernutrition on diaphragm fiber size, SDH activity, and fatigue resistance. *J. Appl. Physiol.* 66 2196–2205. 10.1152/jappl.1989.66.5.2196 2745285

[B120] Sieck G. C. Sacks R. D. Blanco C. E. Edgerton V. R. (1986). SDH activity and cross-sectional area of muscle fibers in cat diaphragm. *J. Appl. Physiol.* 60 1284–1292. 10.1152/jappl.1986.60.4.1284 2939051

[B121] Sieck G. C. White T. A. Thompson M. A. Pabelick C. M. Wylam M. E. Prakash Y. S. (2008). Regulation of store-operated Ca2+ entry by CD38 in human airway smooth muscle. *Am. J. Physiol. Lung. Cell Mol. Physiol.* 294 L378–L385. 10.1152/ajplung.00394.2007 18178673

[B122] Sieck G. C. Zhan W. Z. Prakash Y. S. Daood M. J. Watchko J. F. (1995). SDH and actomyosin ATPase activities of different fiber types in rat diaphragm muscle. *J. Appl. Physiol.* 79 1629–1639. 10.1152/jappl.1995.79.5.1629 8594023

[B123] Smirnova E. Griparic L. Shurland D. L. van der Bliek A. M. (2001). Dynamin-related protein Drp1 is required for mitochondrial division in mammalian cells. *Mol. Biol. Cell* 12 2245–2256. 10.1091/mbc.12.8.2245 11514614 PMC58592

[B124] Song Z. Ghochani M. McCaffery J. M. Frey T. G. Chan D. C. (2009). Mitofusins and OPA1 mediate sequential steps in mitochondrial membrane fusion. *Mol. Biol. Cell* 20 3525–3532. 10.1091/mbc.E09-03-0252 19477917 PMC2719570

[B125] Trian T. Benard G. Begueret H. Rossignol R. Girodet P. O. Ghosh D. (2007). Bronchial smooth muscle remodeling involves calcium-dependent enhanced mitochondrial biogenesis in asthma. *J. Exp. Med.* 204 3173–3181. 10.1084/jem.20070956 18056286 PMC2150973

[B126] Upton J. P. Wang L. Han D. Wang E. S. Huskey N. E. Lim L. (2012). IRE1alpha cleaves select microRNAs during ER stress to derepress translation of proapoptotic Caspase-2. *Science* 338 818–822. 10.1126/science.1226191 23042294 PMC3742121

[B127] van Vliet A. R. Verfaillie T. Agostinis P. (2014). New functions of mitochondria associated membranes in cellular signaling. *Biochim. Biophys. Acta* 1843 2253–2262. 10.1016/j.bbamcr.2014.03.009 24642268

[B128] Vandewynckel Y. P. Laukens D. Geerts A. Bogaerts E. Paridaens A. Verhelst X. (2013). The paradox of the unfolded protein response in cancer. *Anticancer Res.* 33 4683–4694. 24222102

[B129] Vannuvel K. Renard P. Raes M. Arnould T. (2013). Functional and morphological impact of ER stress on mitochondria. *J. Cell Physiol.* 228 1802–1818. 10.1002/jcp.24360 23629871

[B130] Verfaillie T. Rubio N. Garg A. D. Bultynck G. Rizzuto R. Decuypere J. P. (2012). PERK is required at the ER-mitochondrial contact sites to convey apoptosis after ROS-based ER stress. *Cell Death Differ.* 19 1880–1891. 10.1038/cdd.2012.74 22705852 PMC3469056

[B131] White T. A. Xue A. Chini E. N. Thompson M. Sieck G. C. Wylam M. E. (2006). Role of transient receptor potential C3 in TNF-alpha-enhanced calcium influx in human airway myocytes. *Am. J. Respir. Cell Mol. Biol.* 35 243–251. 10.1165/rcmb.2006-0003OC 16574942 PMC2643259

[B132] Wright D. B. Trian T. Siddiqui S. Pascoe C. D. Johnson J. R. Dekkers B. G. (2013a). Phenotype modulation of airway smooth muscle in asthma. *Pulm. Pharmacol. Ther.* 26 42–49. 10.1016/j.pupt.2012.08.005 22939888

[B133] Wright D. B. Trian T. Siddiqui S. Pascoe C. D. Ojo O. O. Johnson J. R. (2013b). Functional phenotype of airway myocytes from asthmatic airways. *Pulm. Pharmacol. Ther.* 26 95–104. 10.1016/j.pupt.2012.08.003 22921313

[B134] Yap J. Chen X. Delmotte P. Sieck G. C. (2019). TNFα selectively activates the IRE1α/XBP1 endoplasmic reticulum stress pathway in human airway smooth muscle cells. *Am. J. Physiol. Lung. Cell Mol. Physiol.* (in press). 10.1152/ajplung.00212.2019 PMC7099431 31940218

[B135] Yoshida H. Matsui T. Yamamoto A. Okada T. Mori K. (2001). XBP1 mRNA is induced by ATF6 and spliced by IRE1 in response to ER stress to produce a highly active transcription factor. *Cell* 107 881–891. 10.1016/s0092-8674(01)00611-610 11779464

[B136] Youle R. J. van der Bliek A. M. (2012). Mitochondrial fission, fusion, and stress. *Science* 337 1062–1065. 10.1126/science.1219855 22936770 PMC4762028

[B137] Yu Z. H. Wang Y. X. Song Y. Lu H. Z. Hou L. N. Cui Y. Y. (2013). Up-regulation of KCa3.1 promotes human airway smooth muscle cell phenotypic modulation. *Pharmacol. Res.* 77 30–38. 10.1016/j.phrs.2013.09.002 24055799

[B138] Zeeshan H. M. Lee G. H. Kim H. R. Chae H. J. (2016). Endoplasmic reticulum stress and associated ROS. *Int. J. Mol. Sci.* 17:327. 10.3390/ijms17030327 26950115 PMC4813189

[B139] Zeng L. Zampetaki A. Margariti A. Pepe A. E. Alam S. Martin D. (2009). Sustained activation of XBP1 splicing leads to endothelial apoptosis and atherosclerosis development in response to disturbed flow. *Proc. Natl. Acad. Sci. U.. A.* 106 8326–8331. 10.1073/pnas.0903197106 19416856 PMC2676169

[B140] Zuo L. Otenbaker N. P. Rose B. A. Salisbury K. S. (2013). Molecular mechanisms of reactive oxygen species-related pulmonary inflammation and asthma. *Mol. Immunol.* 56 57–63. 10.1016/j.molimm.2013.04.002 23665383

